# Morphological, Electrical, and Chemical Characteristics of Poly(sodium 4-styrenesulfonate) Coated PVDF Ultrafiltration Membranes after Plasma Treatment

**DOI:** 10.3390/polym11101689

**Published:** 2019-10-15

**Authors:** Ivette G. Sandoval-Olvera, Pilar González-Muñoz, Darío R. Díaz, Ángel Maroto-Valiente, Nelio A. Ochoa, Francisco J. Carmona, Laura Palacio, José I. Calvo, Antonio Hernández, Mario Ávila-Rodríguez, Pedro Prádanos

**Affiliations:** 1 Departamento de Química, Universidad de Guanajuato, Cerro de la Venada s/n, Guanajuato 36040, Gto., Mexico; 2 Laboratorio de Membranas y Biomateriales, Instituto de Física Aplicada, Universidad Nacional de San Luis-CONICET, Chacabuco 917, San Luis D5700BWS, Argentina; 3 Departamento de Química Inorgánica y Química Técnica, Facultad de Ciencias, Universidad Nacional de Educación a Distancia (UNED), 28040 Madrid, Spain; 4 Dpto. Física Aplicada. Escuela Politécnica, Universidad de Extremadura, 10004 Cáceres, Spain; 5 Grupo de Superficies y Materiales Porosos, Dpto. Física Aplicada, Facultad de Ciencias, Universidad de Valladolid, 47071 Valladolid, Spain; 6 Istitute of Sustainable Processes, Dr. Megelina s/n, 47011 Valladolid, Spain

**Keywords:** UF membrane modification, membrane characterization, electrical impedance spectroscopy, AFM, LLDP, XPS

## Abstract

A commercial ultrafiltration (UF) membrane (HFM-183 de Koch Membrane Systems) made of poly(vinylidene fluoride) (PVDF), was recovered with a negatively-charged polyelectrolyte (poly(sodium 4-styrenesulfonate)) (PSS), and the effects on its electric, chemical, and morphological properties were analyzed. Atomic force microscopy (AFM), liquid–liquid displacement porometry, Electrical Impedance Spectroscopy, X-ray photoelectron spectroscopy, and Raman spectroscopy were used to investigate the modifications induced by the deposition of PSS on the PVDF positively-charged membrane and after its treatment by a radio frequency Ar-plasma. These techniques confirmed a real deposition and posterior compaction of PSS with increasing roughness and decreasing pore sizes. The evolution of the electric resistances of the membranes confirmed crosslinking and compaction with shielding of the sulfonated groups from PSS. In this way, a membrane with a negatively-charged active layer and a pore size which was 60% lower than the original membrane was obtained. The composition of the additive used by manufacturers to modify PVDF to make it positively charged was obtained by different procedures, all of which depended upon the results of X-ray photoelectron spectroscopy, leading to fairly consistent results. This polymer, carrying positive charges, contains quaternary nitrogen, as confirmed by XPS. Moreover, Raman spectroscopy confirmed that PVDF changes from mostly the β to the α phase, which is more stable as a substrate for the deposited PSS. The aim of the tested modifications was to increase the retention of divalent anions without reducing permeability.

## 1. Introduction

Poly(vinylidene fluoride) (PVDF) membranes are frequently used for ultrafiltration and nanofiltration due to their good proprieties, including high mechanical and chemical resistances and thermal stability [1]. In order to increase their functionality, their proprieties of hydrophilicity and fouling resistance can be improved by modifying their surfaces [2,3,4,5,6]. Some work has also been devoted to plasma-assisted grafting [7,8,9]. Both these approaches are very promising; therefore, the combination of these techniques may lead to an improvement and consolidation of the surface properties of PVDF membranes without the loss of their good properties. Recently, this procedure was followed with commercially-available ultrafiltration (UF) membranes made with PVDF (HFM-183, Koch Membrane Systems, Wilmington, MA, USA) by depositing sodium polystyrene sulfonate (PSS) and ulterior plasma treatment, leading to good chromate and phosphate retention [10]. Here, this modification procedure will be comprehensively characterized using many complimentary techniques applied to both the pristine and modified membranes to understand how these modifications cause retention improvements. Specifically, pristine and modified membranes will be characterized, paying special attention to their electric properties, as revealed by impedance spectroscopy (EIS). The porous structure of the membranes will be analyzed using liquid–liquid displacement porosimetry (LLDP). Finally, their surfaces will be studied, paying special attention to their morphology by atomic force microscopy (AFM) and their chemistry using X-ray photoelectron spectroscopy (XPS) and Raman spectroscopy.

The charge on the membrane surface has a considerable effect on its separation mechanism, especially when dealing with nanofiltration membranes. Recently, electric impedance spectroscopy, (EIS) was used to investigate the electrical conductivity of membranes provided with charged functional groups. For example, Roghmans et al. [11] studied the ionic selectivity of membranes modified by recovering them with several microgels. According to their results, the membranes covered by neutral microgels showed properties which were close to those of unmodified membranes and compatible with diffusion-like behavior at low frequencies. Those membranes modified with charged microgels acquired higher resistances because cations were retained to some extent. High frequency lobes appeared for the membranes when immersed in monovalent ions; Roghmans et al. attributed this to concentration polarization in the membrane-modification interface. They observed that the frequency of these arcs decreased with increasing valences of the ions, showing that divalent ions transport more slowly than monovalent ones.

Liquid–liquid displacement porosimetry (LLDP) is a very precise technique to elucidate pore radii, pore density, and molecular weight cut-off. Calvo et al. [12,13,14] compared the LLDP outcomes with those of computerized image analyses from scanning electron microscopy (SEM-CIA) for UF membranes, showing a fair degree of accordance between both these methods. They also evaluated the molecular weight cut off (MWCO) for these membranes from cumulative pore size distributions obtained by LLDP.

Atomic force microscopy (AFM) is a technique of scanning probe microscopy that reveals surface topography by the interaction of a sharpened tip with the surface [15]. Changes detected by AFM provide information on the deposition onto, and removal from, membrane surfaces. For example, Rashdi et al. [16] used nanofiltration membranes (NF270) to separate heavy metals ions. Using AFM, they analyzed the roughness and correlated its decrease with the deposition of metal hydroxides and fouling. Kulikov et al. [17] reported on the formation of aggregates of polycarbodiimide-*g*-polystyrene and the reordinations induced in the polymer that they detected using tapping AFM. 

X-ray photoelectron spectroscopy (XPS) gives important information on the chemical composition of a surface by analyzing peaks for different bonding energies [18]. Wei et al. [19] performed a plasma treatment with carbon tetrafluoride on poly(ether sulfone), and studied the induced changes by XPS. They detected an increase in the fluoride content on the surface of the membranes. Baroña et al. [20] incorporated aluminosilicate, single-walled nanotubes (SWNTs) into thin film nanocomposite (TFN) membranes. Using XPS, they quantified the amounts of aluminum and silica incorporated onto the membrane surface.

Khulbe and Matsuura [21] reviewed the potential of Raman Spectroscopy to study synthetic membranes. They showed that Raman Spectroscopy is useful to clarify both the inter- and intra- molecular interactions between functional groups, to analyze the crystalline structure, to study the changes in the polymer structure during membrane formation or modification, and to study coated membranes. Recently, Virtanen et al. [22] showed the utility of Raman Spectroscopy for foulant characterization and online monitoring. Keen et al. [23] used mapping Raman Spectroscopy to analyze a polypropylene/polyethylene copolymer blended with small amounts of ethylene-propylene rubber appearing as micrometer domains that, after Ar-plasma treatment, increased in size because of their resistance to damage due to crosslinking. Dufour et al. [24] analyzed PVP profiles on PVDF hollow fibers by confocal Raman Spectroscopy.

Here, it will be shown that PSS recovering and the plasma treatment of positively-charged PVDF membranes improve its properties substantially. These properties, including chemical, electrical, and morphological ones, will be studied by an innovative array of methodologies. In summary, a method to obtain new, stable, and good perm-selective membranes by simple, less-reported modification technologies of commercial membranes will be confirmed.

## 2. Material and Methods

### 2.1. Materials and Chemicals

UF membranes made with poly(vinylidene fluoride) (PVDF) (HFM-183, Koch Membrane Systems, Wilmington, MA, USA), henceforth HFM-183, were chosen as the starting membranes. According to the manufacturer, these membranes are positively charged and have a molecular weight cut-off (MWCO) of 100 kDa. 

These membranes were modified using poly(sodium 4-styrene sulfonate) (PSS) with a MWCO of 70 kDa. The polymer was bought in 30% aqueous solution from Sigma Aldrich (Sigma-Aldrich, St. Louis, MO, USA). Other chemicals, such as potassium dichromate, potassium chloride, sodium hydroxide, hydrochloric acid, isobutanol, glycerol, etc., were bought in analytical grade from Sigma-Aldrich (St. Louis, MO, USA).as well. Ultrapure (ASTM Type I) water was used.

### 2.2. Membrane Modification

The membranes were modified using a technique previously reported by us [10]. First, HFM-183 discs of a diameter of 50 mm were washed and 50 mL of a 20.8 g·L^−1^ aqueous solution of PSS was filtered immediately under a pressure of 8 bar. A dead-end cell from Sterlitech (HP4750, Sterlitech Co., WA, USA) was used. The cell was pressurized with nitrogen, and each membrane disc had an area of 14.6 cm^2^ open to flux. The process was performed without stirring in order to allow uniform coverage to occur. By considering the mass balance, this procedure resulted in a deposition of 71 mg of PSS·cm^−2^ on the membrane. Thereafter, the membranes were immersed in glycerol and dried at 35 °C for one hour. 

Plasma treatment was performed in a radiofrequency plasma chamber (Expanded Plasma Cleaner PDC-001, Harrick Plasma, Ithaca, NY, USA) connected to a flux mixer (PlasmaFlo PDC-FMG, Harrick Plasma, Ithaca, NY, USA) and a vacuum pump. Argon gas was fluxed at 0.30 cm^3^·min^−1^ (volumes at standard temperature and pressure (STP)). A power of 10.2 W was used for 15 min, according to previously performed optimization measurements [10].

Two modifications were completed and will be analyzed here. Initially, the membranes were only covered with PSS (membranes HFM-183+PSS). Afterwards, some of them were additionally treated with an argon plasma (membranes HFM-183+PSS+Ar). The modified membranes exhibited some quite different properties, but kept pore sizes and permeability in the ultrafiltration range. They showed a higher retention of divalent ions approaching them in the nanofiltration range. All samples were washed with deionized water by filtration and dried before the various analyses. Washing was stopped when no traces of glycerol or any other contaminant were detected by HPLC analysis of the permeate. In Table 1, some of the functional characteristics of the original and modified membranes are shown.

### 2.3. Membrane Characterization.

Pristine and HFM-183 membranes, along with the corresponding modified membranes (HFM-183+PSS and HFM-183+PSS+Ar), were characterized using several techniques, referred to below, focusing on the surface chemical physics and electrical properties.

#### 2.3.1. Atomic Force Microscopy

AFM images were obtained using a Nanoscope Multimode IIIa^®^ from Digital Instruments (Veeco Metrology Inc., Santa Barbara, CA, USA) in the tapping mode, in accordance with the methods described elsewhere [25]. Roughness and power spectral density (PSD) were analyzed using the NanoScope Software Version 5.30 (Veeco Metrology Inc., Santa Barbara, CA, USA).

#### 2.3.2. Liquid-liquid Displacement Porometry

The distributions of pore sizes were measured by liquid-liquid displacement (LLDP), using a device developed by Calvo et al. [26]. The pores were firstly filled with a liquid which was substituted by another immiscible liquid which entered increasingly wider pores as the pressure increased. In our case, a dilute aqueous solution of isobutanol was used as the initial pore-filling liquid, while an isobutanol-rich water solution pushed it out of the pores. Both solutions were made by mixing 1:1 *w*/*w* water and isobutanol and allowing the solution to separate overnight. The membrane was filled by keeping it under vacuum and immersed in the water-rich solution for ½ hour. Membrane discs of a diameter of 50 mm were measured in triplicate.

Pushing pressure and pore radius are correlated with the cantor equation, provided that the contact angle in the liquid-liquid-membrane interface can be taken as nil [26]:

(1) $$\Delta p=\frac{2\gamma }{r}$$

where γ is the surface tension. If the geometry of the pores is assumed, Δp can be correlated with the number of pores, with each pore radius, n, opened at each Δp. For cylindrical pores, according to the Hagen-Poiseuille equation, the volume flux, $q_{i}$, passing through all the pores opened until $\Delta p_{i}$ is [26]:

(2) $$q_{i}=\sum_{k=1}^{i}\frac{\pi n_{k}r_{k}^{4}\Delta p_{i}}{8\eta l}$$

where η is the viscosity of the pushing liquid and l is the pore length, which is approximately equal to the membrane thickness.

#### 2.3.3. Electrical Impedance Spectroscopy

Electrical Impedance Spectroscopy (EIS) experiments were performed with membrane samples immersed in a water solution of K_2_CrO_4_ (from 0.005 to 0.287 mol L^−1^) at pH = 8 in a measuring cell described elsewhere [27]. An electrical impedance analyzer, Solartron 1260 (Ametek, Berwyn, PA, United States), was used at frequencies from 10 MHz to 10 mHz and with under 10 mV applied AC voltage, at 298 ± 1 K. 

In order to avoid the influence of the electric noise caused by the connections, and of the porous matrix of the membrane, an Open/Short/Load correction was performed, as described elsewhere [28].

#### 2.3.4. X-Ray Photoelectron Spectroscopy

The chemical composition of the membrane surface was analyzed using X-ray photoelectron spectroscopy (XPS) with an ESCA 5701 (Physical Electronics, Chanhassen, MN, USA) with a monochromatic Mg Kα X-ray source (300.0 W, E = 1253.6 eV).

#### 2.3.5. Raman Spectroscopy

The chemical analysis was complemented using a Microscope DXR Raman of Thermo Scientific (Thermo Fisher Scientific, Waltham, MA, USA); a 780 nm laser was used with an energy of 20 mW. Spectra were collected with exposure times of 2 s, and 32 exposures with a 50× objective.

## 3. Results and Discussion

### 3.1. Atomic Force Microscopy.

**Figure 1:**
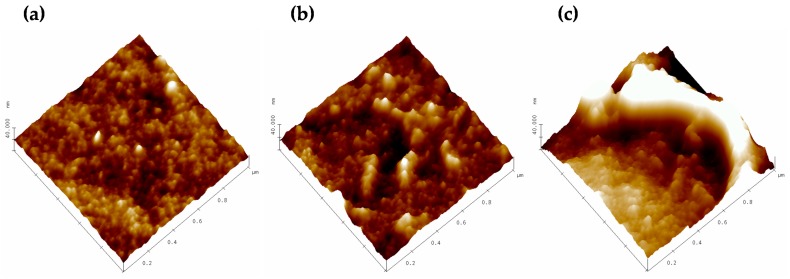
Topographic 1 × 1 μm images of: (**a**) HFM-183, **(b**) HFM-183+PSS, and (**c**) HFM-183+PSS+Ar.

In Figure 1, Atomic Force Microscopy (AFM) images of pristine, modified, and plasma-treated membranes are shown for a scanned area of 1 × 1 μm.

An analysis of many topography images for the scanned areas from 0.5 μm × 0.5 μm to 5 μm × 5 μm shows that the unmodified membranes are the flattest, with more regular surface structures. When recovered with PSS, the surface becomes more irregular, with some clusters. Finally, irregularities increase after the plasma treatment due to a possible roughing down of the deposited PSS that was not totally anchored onto the surface.

**Figure 2:**
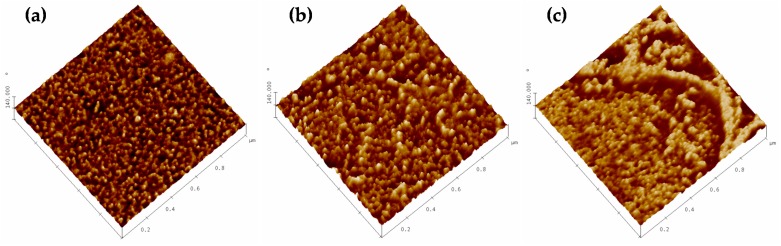
Phase contrast images for 1 × 1 μm scanned areas of (**a**) HFM-183, (**b**) FM-183+PSS, and (**c**) HFM-183+PSS+Ar.

Phase contrast images reveal changes in the viscoelastic properties of the surface or sharp morphologic changes. Figure 2 shows phase contrast images for the three membranes studied (the same samples as those in in Figure 1). There, it appears clear that modified membranes show more phase changes, especially for the plasma-treated membrane.

**Figure 3:**
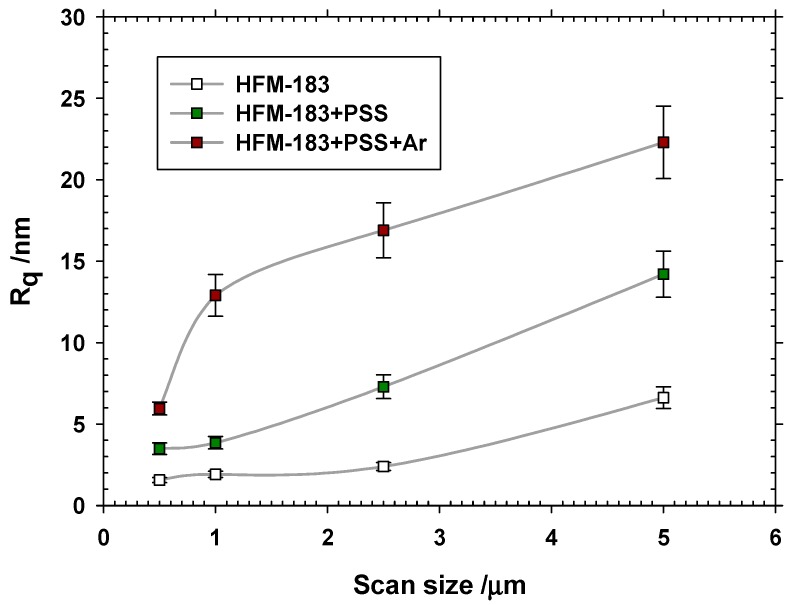
Roughness versus scan size.

Roughness can be characterized by the root mean square (RMS) roughness, $R_{q}$:

(3) $$R_{q}=\sqrt{\frac{\sum z_{i}^{2}}{N}}$$

where $z_{i}$ is the height of the i-th pixel and N is the total number of pixels. Figure 3 shows roughness as a function of the scanned area with the corresponding error bars evaluated for 5 measurements each.

It can be seen that the pristine HFM-183 membrane is quite flat, and that roughness increases with each successive modification step. Probably, PSS deposition is not totally uniform, and plasma treatment crosslink and partially damage some of the PSS chains on the membrane surface.

It is common to see roughness increasing with scanned area; this issue can be studied using the Power Spectral Density (PSD). This function refers to the roughness amplitude versus its space frequency. It allows an appropriate filtering of the signal to be undertaken in order to eliminate unwanted noise. For example, a high frequency corresponds to image acquisition noise, while low frequencies correspond to scratches and cracks.

The isotropic PSD, for a digitalized image of a line of length L, that in fact consists of *N* points spaced at distance $d_{0}$, can be evaluated by [29]:

(4) $$PSD=\frac{d_{0}^{2}}{\pi (m-1)}{\left(\sum_{n=1}^{N}e^{i\frac{2\pi }{N}\left(n-1)(m-1)\right)}z_{n}\right)}^{2}$$

where *m* is a function of space frequency.

(5) $$f=\frac{m-1}{Nd_{0}}$$

where $i=\sqrt{-1}$ and frequencies go from 1/L to N/2L. It is more convenient to use this 1D PSD, because scanning is performed following lines, thus moving along the x axis much faster than along the y axis. Irrelevant noise has been removed by subtracting a 0 μm × 0 μm scan from all pixels in each real scan, performed with the same parameters as each finite size scan.

**Figure 4:**
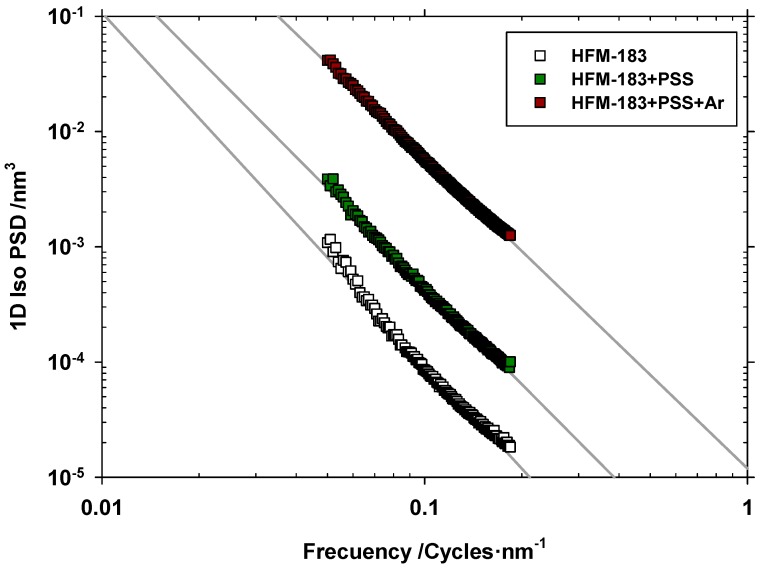
One dimensional PSD versus space frequency.

A double logarithmic plot of the one-dimensional PSD versus the space frequency usually yields a linear plot in the middle range of frequencies. This reveals an auto-similarity of the actual surface structure which is neither conditioned by the image acquisition noise nor by cracks. One such plots are presented in Figure 4 for the original and both modified membranes. The fractal dimension of the sample can be evaluated from the slopes, α, of the straights such as [30]:

(6) $$D_{f}^{1D}=\frac{5+\alpha }{2}$$

The corresponding values of $D_{f}^{1D}$ and roughness, $R_{q}^{fr}$, are shown in Table 2. Note that the obtained roughness is always smaller than those shown in Figure 3, because some components of roughness that do not correspond to the relevant features of the surface morphology have been removed. Note that the roughness increases after recovering and plasma treatment.

Turning to the fractal dimension, it seems that the explored lines through the pristine membranes are almost fully linear (one-dimensional). The dimension of such lines increases after PSS recovery, and long space wavelengths appear (see Figure 4). Finally, after plasma treatment, shorter space wavelengths appear (see again Figure 4), with a slightly higher fractal dimension. This should be attributed to partial PSS-crosslinking or devastation after the plasma treatment.

### 3.2. Liquid-liquid Displacement Porometry

**Figure 5:**
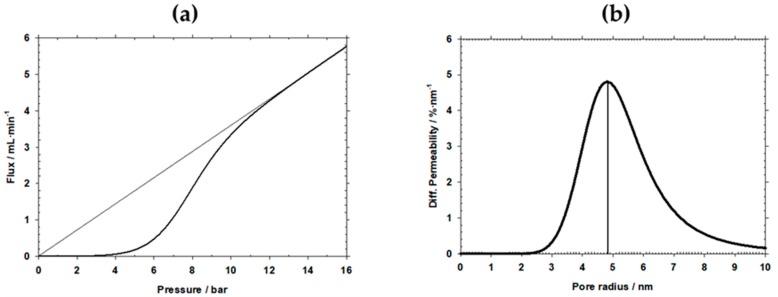
Flux versus pressure fort the HFM-183 membrane **(a)** and the corresponding permeability pore size distribution **(b)**.

Pore sizes of HFM-183, HFM-183+PSS, and HFM-183+PSS+Ar were measured by LLDP. An example of the flux versus pressure and the corresponding permeability distribution for pore sizes for the HFM-183 membrane is shown in Figure 5.

In Table 3, the mean pore radii, *r*_p,_ according to the corresponding pore size distributions are shown, along with their standard deviations and the molecular weight cut-off (MWCO) evaluated from them, as described by Calvo et al. [26]. Note that these radii are comparable to those obtained from image analysis of the SEM pictures shown in Table 1.

Table 3 presents the pore sizes for the pristine and modified membranes. The pore size of HFM-183 membranes are reduced by half after their modification, although MWCO reduced from approximately 50 kDa to around 11 kDa after modification. Note that the small differences between the PSS recovered membranes and those that were plasma treated afterwards are well within the error range. The similarity of the LLDP and SEM pore sizes implies that the most restrictive section (LLDP) along the pores is on the surface (SEM) of the membrane.

### 3.3. Electrical Impedance Spectroscopy

**Figure 6:**
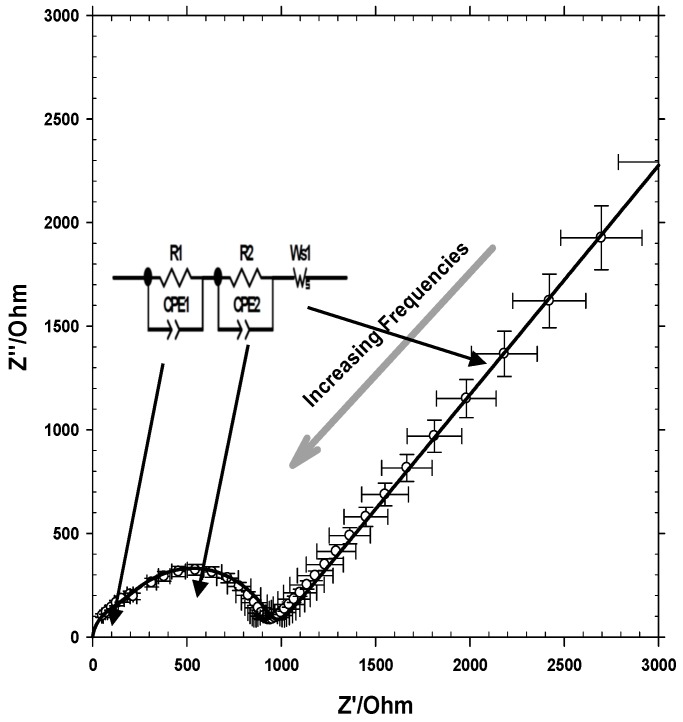
Nyquist plot for the HFM183 membrane and a concentration of 0.005 mol·L^−1^ of K_2_CrO_4_. Experimental points with error crosses and the best fitted curve are shown.

EIS has made possible the electrical characterization of our original and modified membranes when K_2_CrO_4_ solutions in several concentrations fill their pores. In Figure 6, we show an example of the Nyquist plot for the HFM-183 membrane. It appears as a flattened lobe with some asymmetry, followed by a linear increase of imaginary versus real impedance for low frequencies with a slope near 1. The first asymmetric lobe may consist of two overlapping lobes, and can be associated with the equivalent circuits, as shown in Figure 6, consisting of parallel constant phase elements (CPE) plus resistance (R) circuits. A finite length Warburg (FLW) element appears at the lowest frequencies measured, giving a 45° slope, as shown in Figure 6.

The first R-CPE element, R1-CPE1, should correspond to the zone where the ions move more easily, i.e., the pores of the membrane support. They might correspond to the highest frequencies, i.e., to the left of the Figure 6. When ions penetrate the narrower pores in the active layer, they respond to lower frequencies, or longer relaxation times, appearing on the right side of the lobe in Figure 6. The FLW circuits describe diffusion which is associated with charge-transfer resistance or double layer capacitance. In our case, Ws1 should describe the electric double layer appearing on the Hg electrode. The fitting, as shown in Figure 6, was quite fair.

**Figure 7:**
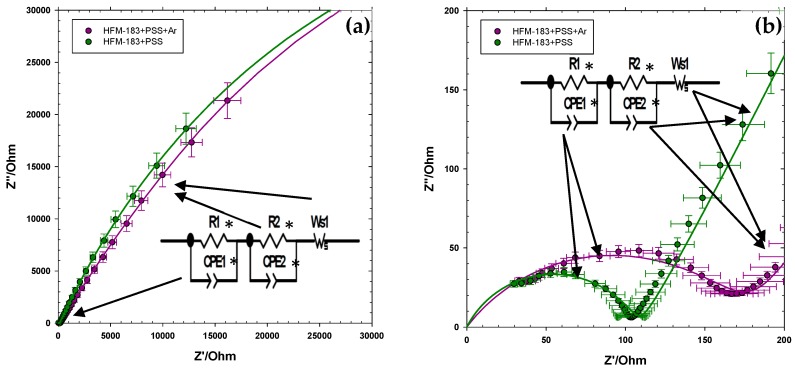
Nyquist plot for the HFM-183+PSS and HFM-183+PSS+Ar membranes and a K_2_CrO_4_ concentration of 0.005 mol·L^−1^. Complete plot from 10 MHz to 10 MHz **(a)** and high frequency zoom **(b)**.

The Nyquist plot changes when the membrane is recovered with PSS, as seen in Figure 7, where the plot is shown for the 0.005 mol·L^−1^ concentration of K_2_CrO_4_. It is apparent that within the low frequency zone, there is a non-linear dependency with an initial slope of over 45° (Figure 7a); we will assume that this is the beginning of the low-frequencies-lobe. On the other hand, in the high frequency range, a flattened lobe appears that should correspond to the convolution of the support one with some (impossible to separate) contribution of a small penetration of PSS into some of the pores of the active layer (Figure 7b).

**Figure 8:**
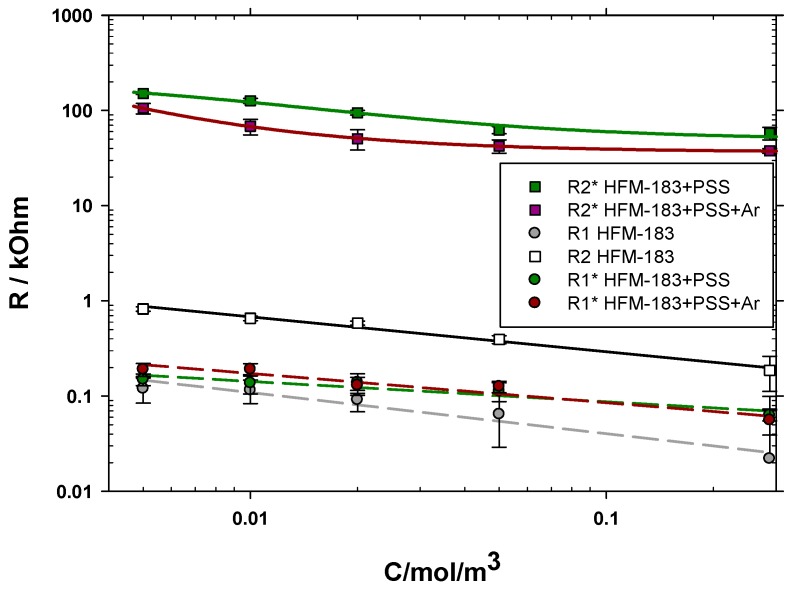
Resistances for the pristine membrane, the PSS recovered one, and the plasma-treated one are shown as a function of the K_2_CrO_4_ concentration.

The dependency of the support and active layer resistances with concentration is shown in Figure 8. Note that R1* and R2* refer to the PSS recovered or the plasma-treated membrane. In both cases, number 1 refers to the part of the membrane (including the support and active layer) which was not affected by the modification. It is worth mentioning that we assumed that the FLW (Ws1) for the modified membranes was equal to that of the pristine one in order to fit the resistances. Note that the electrode double layer should be independent of the membrane itself. Much bigger errors, with similar resistances, would be obtained if Ws1 were kept as a fitting parameter.

All resistances decrease with increasing concentrations. The resistances for the support layers R1 and R1* are always lower. In all cases, the resistances for the support layer are quite similar. The R2* resistance of the HFM-183+PSS+Ar membrane is slightly lower than that of the HFM-183+PSS membrane. This could be attributed to a partial removal of PSS from inside the active layer pores due to the plasma treatment. In both modified membranes, R2* is higher than R2, which could be due to the negative character of PSS and the induced decrease in porosity that, together, would hinder the freedom of chromate ions inside the active layer pores; this would cause a Donnan exclusion potential between the PSS recovered zones (negative) and those (close to the support layer) where PSS did not arrive, and keep their original positive charges. 

The lower active layer resistance for HFM-183+PSS+Ar agrees with the lower zeta potential (lower negative charge), as shown elsewhere [10] (see Table 1). 

As mentioned, the resistances through the porous support are quite similar to each other, and they should correspond to conductivities which are close to that of the free solution, due to the wide (from 0.25 to tens of μm) and neutral pores that they cross there. In order to address this issue, we can evaluate the Δx/θ from R1 and Equation (7) [27]:

(7) $$\kappa =\frac{1}{R1}\frac{\Delta x}{A\theta }$$

where A is the area of the membrane. Here, we assumed that the mobility within the support layer is, in effect, quite similar to that of the free solution [27]. Once Δx/θ is known, we can determine the solution dielectric constant:

(8) $$\varepsilon =C1\frac{\Delta x}{A\theta \varepsilon_{0}}$$

in terms of the capacity of the active layer C1. Here, $\varepsilon_{0}$ is the vacuum permittivity. The so obtained value for ε is 0.83 ± 0.15, that, considering the error range, corresponds to the water solution typical value. This agreement has already been shown for this kind of system [27].

In order to determine the conductivity of the active layer, which is clearly more adequate as far as the influence of the possible variations of porosity are concerned, we can use Equation (8), as done for the support layer, once Δx/θ are known. The thickness versus porosity quotient, Δx/θ, can be obtained from Hagen-Poiseuille theory (assuming cylindrical pores), the experimental permeabilities, $L_{p}=q/\Delta p$ [10] (Table 1), and the pore size obtained by liquid–liquid porometry (Table 3):

(9) $$\frac{\Delta x}{\theta }=\frac{r_{p}^{2}}{8\eta L_{p}}$$

Note, when comparing with Equation (2), that $\theta =n\pi r_{p}^{2}$, with n being the total number of pores per unit area, all the pores are assumed to have the same pore radius $r_{p}$.

**Figure 9:**
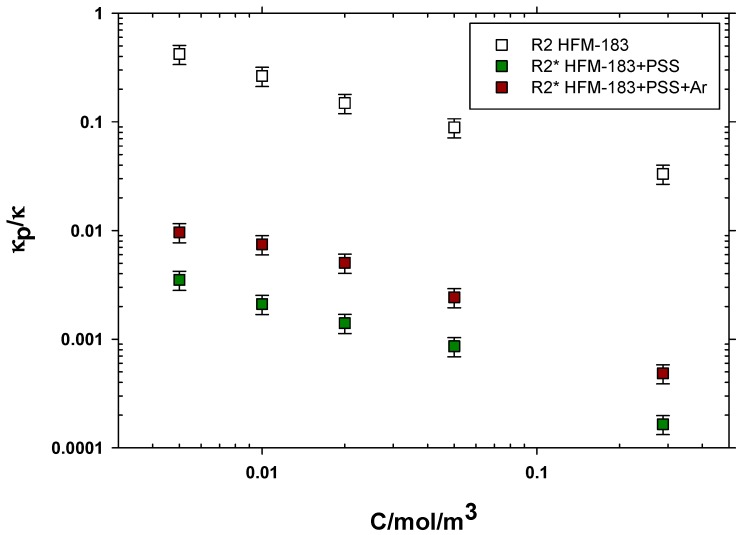
Conductivity through the active layer pores relative to the free solution conductivity as a function of K_2_CrO_4_ concentration for the HFM-183, HFM-183+PSS, and HFM-183+PSS+Ar membranes.

The conductivity inside the pores of the active layer of the pristine and modified membranes are shown divided by the free solution conductivity, as evaluated using the work of Iadicicco et al. [31], in Figure 9. The behavior of such conductivity ratios is analogous to that of the resistances shown in Figure 8. In both cases, most of the influence of concentration appears for low concentrations, with a very fast decrease in resistance and conductivity. Note that both Figure 8 and Figure 9 are double log plots. This is quite common in highly-charged and ion-exchange membranes [32].

### 3.4. X-Ray Photoelectron Spectroscopy, XPS.

**Figure 10:**
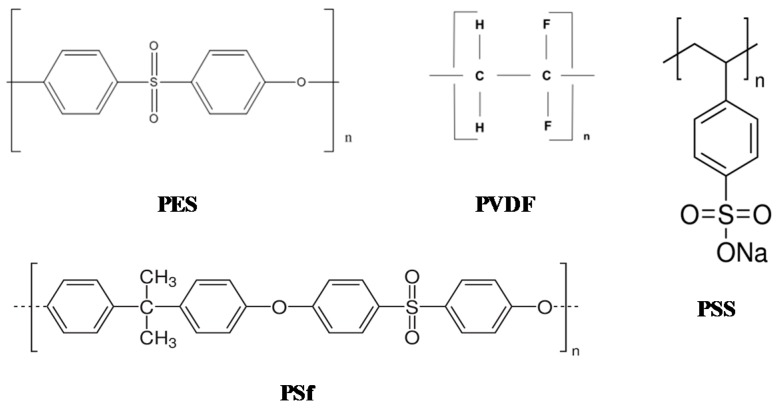
Polymers present in pristine HFM-183 membranes and their modifications, HFM-183+PSS and HFM-183+PSS+Ar.

**Figure 11:**
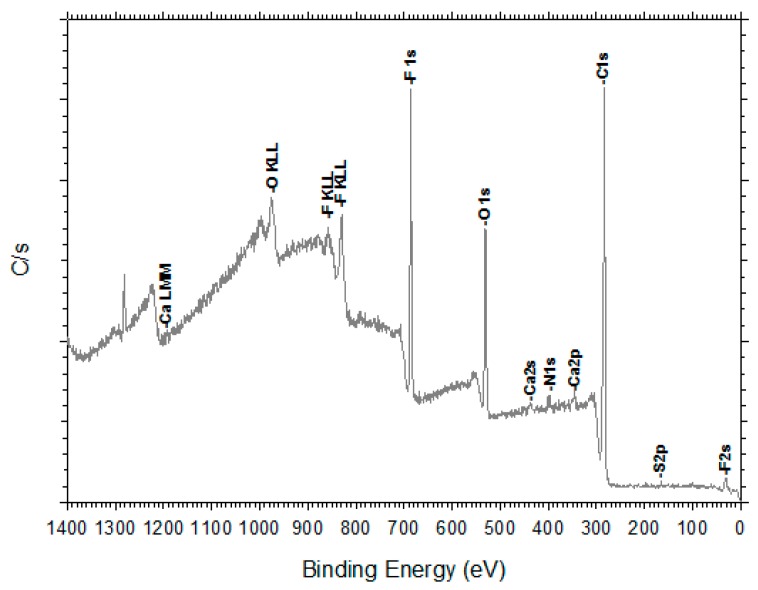
XPS spectrum for the HFM-183 membrane.

The XPS spectra of the pristine HFM-183 membrane and the modified ones (HFM-183+PSS and HFM-183+PSS+Ar) were sampled and studied. Figure 10 shows some of the polymers involved in the analysis of our membranes. In Figure 11, an example of a XPS spectrum for the HFM-183 membrane is shown. The atomic percentages of the unmodified and modified membranes are shown in Table 4.

The HFM-183 membrane, according to the manufacturer, has an active layer consisting of PVDF with a positive charge. However, PVDF should have a C/F ratio of 1 (see Figure 10), but in our case (see Table 4), this ratio is close to 4; thus, other materials must be present. It is worth noting that, as shown elsewhere [10] by us, Energy-Dispersive X-ray Spectroscopy (EDS) reveals higher proportions of S compared to those obtained from XPS; see Table 4. Nevertheless, this could be attributed to the deeper scope reached by EDS and the extra S detected which is attributable to the membrane support. Apart from this S content, there are large amounts of N and relatively large amounts of O and C that could not have penetrated into the support layer. Moreover, if all the sulfur in Table 4, for the HFM-183 membrane, came from the underlying polysulfone and its content of O and C was taken into account, and we considered that all the F came from PVDF with C/F = 1, we can conclude that some additional compound would be needed with C = 73.13%, O = 23.18%, N = 3.27%, and Si = 0.42%. If the support was supposed to be polyethersulfone instead of polysulfone, the elemental percentages of such an additional compound would be quite similar, i.e., C = 73.96%, O = 22.50%, N = 3.134%, and Si = 0.41%. 

Actually, the S2p peak in Table 4 appears at around 166.3 eV for all the pristine and modified membranes, which is compatible with the SO_2_ from polysulfone or polyethersulfone, but it could also come from $-SO_{3}^{-}$ from the sulfonate groups of PSS [33], which would explain its substantial increase for the PSS-containing membranes. 

The composition of the pristine membrane may be explained in terms of grafted compounds with O and N with positive charges due to the presence of quaternary nitrogen. In the literature, some examples can be found that satisfy these requirements. Brite et al. modified a PVDF membrane by grafting tetraethylpentamine (TEPA) by electron beam irradiation [34]. They obtained positively-charged PVDF membranes with a zeta potential that was quite similar to that of HFM-183 membranes due to the presence of quaternary ammonia. Park et al. obtained positively-charged PVDF membranes by grafting positively-charged hyperbranched polyglycerols (PHPGs) with quaternary ammonium groups [33]. In this case, not only the zeta potentials, but also the O/N ratios, are quite similar to those of the pristine HFM-183 membrane.

The HFM-183+PSS membrane shows less F content than HFM-183. This is due to the increase in S, due to the PSS layer (theoretical composition: C = 61.54%, O = 23.08%, S = 7.69%, and Na = 7.69%). If we consider that, in this case, XPS does not penetrate the support layer and C/F = 1 for PVDF, while PSS has its theoretical composition, we can determine that the additive grafted onto PVDF to make it positively charged would be as follows: C = 75.33%, O = 20.05%, N = 2.26%, and Si = 2.36%; this is quite a similar composition to that obtained from the HFM-183 data. The agreement is notable if we take into account the error ranges (~10%) usually found in XPS results. The increase in Si could be a residue or a contaminant introduced during grafting. However, a satellite peak to the primary Si 2p peak usually appears in XPS due to the use of a magnesium anode in the x-ray source [35]. In addition, organic silicon compounds are widely used in the synthesis of polymer materials. This might cause the detected increase of Si content in the samples coated with PPS.

The HFM-183-PSS-Ar membrane presents a significant reduction of fluoride. This may be due to the crosslinking of the PSS chains (induced by plasma) which would compact the surface and hinder the penetration of XPS. If, by applying the same approach as that used for the HFM-183-PSS membrane, we evaluate the composition of the PVDF grafting, we get: C = 75.69%, O = 21.06%, N = 1.87%, and Si = 1.38%. This is, again, quite similar to the results from the data for HFM-183-PSS and HFM-183. The sulfur content in HFM-183-PSS-Ar is quite similar to that of HFM-183-PSS. Nevertheless, it is worth mentioning that zeta potentials for the membrane treated with plasma [10] correspond to a less negative surface charge density. Because sulfur would be responsible for the negative charge, similar amounts of sulfur in HFM-183-PSS and HFM-183-PSS-Ar would require that sulfonate groups were inactivated somehow after the plasma treatment. Such inactivation could be due to crosslinking or compaction, although it could also be possible that positive charges were created in some way, balancing some of the negative charges carried by the sulfonate groups.

**Figure 12:**
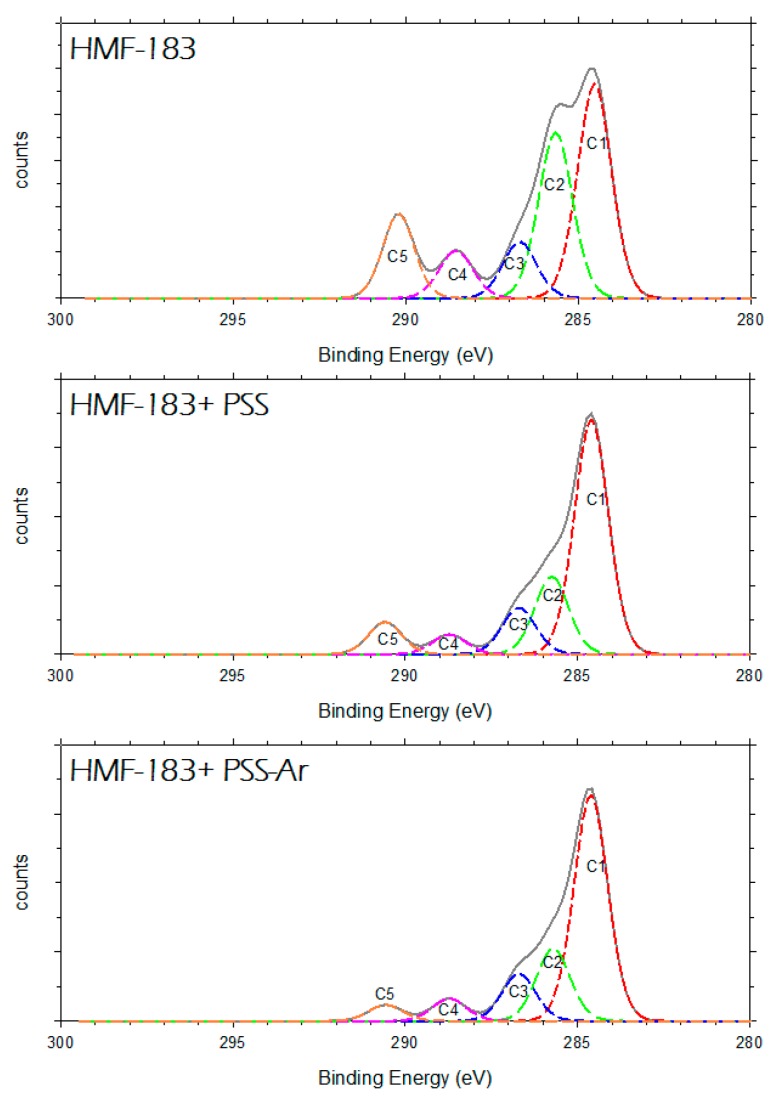
Deconvolution of the C1s peak for the HFM-183, HFM-183+PSS, and HFM-183+PSS+Ar membranes.

The subpeaks within the C1s were fitted and shifted to place the main C1, corresponding to aromatic C, at approximately 284.6 eV. In Figure 12, the fine structure of the C1s peak is shown for the modified and unmodified membranes studied here. In Table 5, the corresponding percentages and positions of the subpeaks are shown. 

The C1 peak may correspond to CH groups (sp^2^ type) or to aromatic carbon [36]. In the pristine membrane, C1 corresponds to 38% total carbon. A small portion could be due to the aromatic rings of polysulfone (or polyethersulfone), and the rest to the possible additive grafted on PVDF (PVDF does not present this signal). The coating of PSS increases C1, probably because of the correspondingly increasing number of aromatic rings. The C2 peak can be assigned to the CH_2_ (sp^3^ type) groups and to the C–N groups [36]. In our case, this peak might appear more intensely for the unmodified membrane, because 50% of PVDF should contribute to this peak (other contributions may come from the carbons and the quaternary ammonium grafted on PVDF). PVDF C2 carbons would constitute 12.5% of C2; the rest of the C2 peak would come from grafting and penetration below the active layer. 

The C3 peak can be assigned to the C–O (alcohols or ethers) and C–S bonds [37]. The original HFM-183 membrane might have alcohol or ether groups that would account for 10% of the C3 carbons. PSS coating should add C–S links, but no any extra C–O bonds. The added C–S bonds would partially mask the preexistent C–O bonds, resulting in a slight increase in the C3 peak. 

The C4 peak, according to the literature, may be attributed to the C–S bonds of the sulfonated benzene rings and to C=O bonds [36,38]. There are fewer C4 carbons after PSS recovery, probably because most of them were in C=O bonds, while the C–S bonds were giving the C3 signal. 

Finally, the C5 peak can be ascribed exclusively to the C–F link from PVDF [36], as shown by the behavior of the peak which is mostly parallel to that of the F peak (see Table 4).

The plasma treatment does not substantially modify the signals of any of these carbon peaks apart from C5 (Figure 12 and Table 5), which declines appreciably for HFM-183+PSS-Ar when compared with HFM-183+PSS. This may be due to compaction by cross-linking, that would make the F atoms less accessible.

Table 6 shows the deconvolution of the N1s peak. The N1 sub-peak at around 399 eV may be attributed to N–H or N–R bonds, while the N2 signal appears at around 402 eV and corresponds to –NR_3_+Cl– or –NH_3_+Cl– links [33]. The relative amounts of these peaks for the pristine HFM-183 membrane are evidence for the presence of quaternary nitrogen.

### 3.5. Raman Spectroscopy.

**Figure 13:**
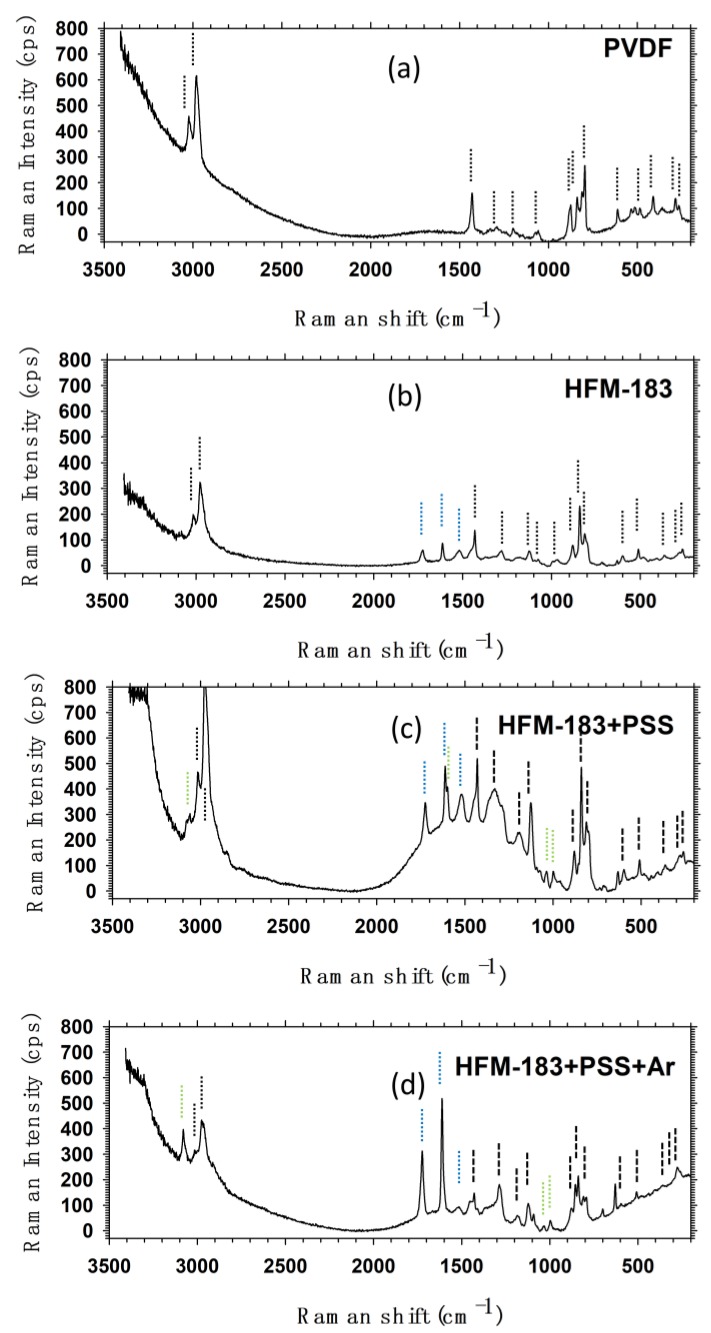
Raman shift for pure PVDF and the membranes studied. (**a**) PVDF pure film, (**b**) HFM-183 pristine membrane, (**c**) HFM-183+PSS membrane, and (**d**) HFM-183+PSS+Ar membrane.

The Raman spectrum for pure PVDF shows characteristic signals in Figure 13, e.g.: A pair C–H stretching signals at 3000 cm^−1^; methylene C–H stretching at 1440 cm^−1^; C–F stretching at 1300 cm^-1^ and 1200 cm^−1^; stretching of the PVDF backbone C–C at 1150 cm^−1^. There are also two bending signals for C–H at 1080 cm^−1^ and 890 cm^−1^. The combination of rocking for methylene and the out-of-phase stretching of the CF_2_ gives the signal at 850 cm^−1^. An intense signal corresponds to the predominance of the β phase (characterized by trans configuration, TTTT) in PVDF. At 810 cm^−1^, the CF_2_ signal corresponds to the *α* phase (characterized by an alternation of trans and gauche links, TGTG) of PVDF. Given that both signals are present, there is a mixture of both phases, although the *β* phase predominates. A twisting (out of phase flexion) or torsion signal for CF_2_ appears at 610 cm^−1^. Other signals corresponding to vibrations of the CF_2_ groups were observed at 510 cm^−1^, 410 cm^−1^, 285 cm^−1^, and 250 cm^−1^.

The HFM-183 membrane spectrum can be compared with the pure PVDF spectrum. Besides the PVDF signals, the HFM-183 membrane shows peaks at 1730 cm^−1^, 1610 cm^−1^, 1520 cm^−1^, and 1000 cm^−1^.

It has been proposed that on PVDF, there are ammonium $-C-NH_{3}^{+}X^{-}$ or imine $R_{1}R_{3}C=NR_{3}^{}$ groups, or both. The stretching signal for the C=N group appears at 1730 cm^−1^; the twisting signal for NH_3_ appears at 1610 cm^−1^; the wagging (in-phase flexion) for the NH_3_ group appears at 1520 cm^−1^; and the stretching of N–H appears at 1000 cm^−1^.

When the membrane is recovered with PSS, seven new signals appear. These new peaks in the Raman spectrum are: the stretching of the aromatic C–H at 3090 cm^−1^; the stretching of the aliphatic C–H bonds at 2900 cm^−1^, which overlaps one of the peaks of PVDF at the same Raman shift; the vibration of the C–H bonds of the methylene group at 1450 cm^−1^; the $-SO_{3}^{-}$ stretching which appears at 1030 cm^−1^; the stretching of C–C of the p-substituted benzene ring gives a signal at 1000 cm^−1^; the stretching of the C–S bond appears at 800 cm^−1^; and the flexion of this group appears at 510 cm^−1^. The Raman spectrum of the HFM-183+PSS membrane confirmed the PSS recovery on the original membrane. Moreover, we can assume that the PSS interactions on the membrane are purely electrostatic, because none of the HFM-183 peaks were changed, and the new ones were those of PSS alone.

The HFM-183+PSS+Ar Raman spectrum showed several peculiarities. The C–H vibration signal at 3000 cm^−1^ that appeared for the HFM-183 membrane and which was also present for the HFM-183+PSS membrane decreased in intensity for the HFM-183+PSS-Ar membrane. The 3100 cm^−1^ (stretching of the aromatic C–H bond, marked with dotted green lines in Figure 13) is more intense for the HFM-183+PSS+Ar than for the untreated HFM-183+PSS. Within the range from 1800–1000 cm^−1^, all peaks, except those at 1600 and 1750 cm^−1^, corresponding to C=N and NH_3_, are less intense. These bonds are probably responsible for the positive charge of the original membrane, and would be partially uncovered by plasma leading to a less negative zeta potential on the membrane surface [10]. The intensity of PVDF peaks within this range and those at 890 cm^−1^, 850 cm^−1^, and 810 cm^−1^ decreased too. The intensity of the 810 cm^−1^ peak was higher than that of 850 cm^−1^. This means that now the α phase dominates over the β phase in PVDF. This change of phase due to energetic radiation has been widely reported in literature [1,39,40]. In the interval from 650 to 250 cm^−1^, the signal at 600 cm^−1^ of the $-SO_{3}^{-}$ group and those of CF_2_ at 410 cm^−1^ and 250 cm^−1^ disappeared.

To summarize, it seems that the plasma treatment induced a change in the PVDF phase from *β* to *α* that could favor PSS stabilization, as suggested by the other characterization techniques used in this study. These configurations are shown in Figure S1 in the Supplementary Material. The loss of some CF_2_ and $-SO_{3}^{-}$ peaks could be due to the crosslinking and etching of some PSS or PVDF chains; the relevant peaks are shown in Table S1 of the Supplementary Material. Finally, zoomed figures showing some detailed peaks are shown in Figure S2 in the Supplementary Material.

## 4. Conclusions

The roughness and fractal dimension increased with the treatments of our membrane, reaching a maximal value after the plasma treatment, as confirmed by AFM. Meanwhile, LLDP indicated a decrease in pore radii and MWCO after the modification of the membrane due to the PSS deposition. A very slight ulterior pore size reduction was caused by the plasma treatment. Both these data correspond to irregular PSS deposition followed by compaction.

The electric resistance (of the more restrictive layer) of the pristine membrane was the lowest, and its conductivity the highest. The plasma-treated membrane showed intermediate resistance and conductivity. This could be attributed to the compaction or crosslinking of PSS with the shielding of the sulfonated groups caused by the plasma treatment. Both modified membranes had higher resistances (and lower conductivities) than the pristine one, which was probably due to the negative character of PSS creating a capacitive double layer at the interface, along with a decrease of pore size and porosity. Moreover, resistances and conductivities were shown to decrease with concentration, as expected. This behavior corresponds to the acquisition of negative charges after PSS coating with a slight decrease in the negative acquired charge due to the crosslinking, compaction, and grinding induced by the plasma treatment. 

XPS probes featured an additional polymer added to the PVDF matrix on the pristine membranes. Different procedures make it possible to calculate the composition of this additional polymer, leading to similar figures (namely: C 73–76%; O 20–23%; N 2–3%; Si 0–2%). This added nitrogen could be quaternary, and was likely be responsible for the positive charge that was observed in the pristine membrane.

Finally, Raman spectroscopy proved that the plasma treatment led to a change in the PVDF configuration, i.e., from β to α, which stabilized the layered, modified membrane. The loss of some CF_2_ and $-SO_{3}^{-}$ peaks suggests the action of crosslinking and etching on PSS (and PVDF) on the membrane active layer.

All these data deepen our understanding of the mechanism of modification induced on the original HFM-183 membrane, and how their charges and retention change.

## Figures and Tables

**Table 1:** Characteristics of the original and modified membranes [10].

Membrane	Water Permeability (m·Pa^−1^·s^−1^)	Pore Radii from SEM Images (r_p_ ± *σ*) (nm)	Observed KCrO4 Retention (%)	Zeta Potential at pH = 7 (mV)
HFM-183	(8.09 ± 1.57)·10^−10^	4.45 ± 0.15	<2	+2.2 ± 0.4
HFM-183+PSS	(1.31 ± 0.10)·10^−10^	2.90 ± 0.20	(58.4 ± 3.5)	−7.8 ± 0.8
HFM-183+PSS+Ar	(0.65 ± 0.01)·10^−10^	2.70 ± 0.25	(66.0 ± 1.4)	−4.3 ± 0.7

**Table 2:** Roughness, *α* slopes, and corresponding $D_{f}^{1D}$.

	$R_{q}^{fr}\;(\mathrm{nm})$ (Figure 4)	*α* (Slopes in Figure 4)	$D_{f}^{1D}$ (Equation (6))
HFM-183	0.490	−2.96	1.02
HFM-183+PSS	0.602	−2.77	1.12
HFM-183+PSS+Ar	1.44	−2.65	1.17

**Table 3:** Pore radii and MWCO according to LLDP.

Membrana	(*r*_p_ ± *σ*) nm	(MWCO ± *σ*) kDa
HFM-183	4.76 ± 0.49	50.49 ± 8.61
HFM-183+PSS	2.37 ± 0.13	11.89 ± 0.31
HFM-183+PSS+Ar	2.31 ± 0.12	11.23 ± 0.99

**Table 4:** Atomic percentages obtained by XPS.

Composition (atom %).	C1s	N1s	O1s	F1s	Na1s	Si2p	S2p
HFM-183	66.01	2.01	14.91	16.65	0	0.26	0.16
HFM-183+PSS	68.79	1.51	17.26	8.14	1.42	1.58	1.29
HFM-183+PSS+Ar	71.1	1.38	19.74	4.04	1.32	1.02	1.39

**Table 5:** Deconvolution of the C1s peak. B.E. stands for binding energy.

	HFM-183		HFM-183+PSS		HFM-183+PSS+Ar	
Component of the C1s Peak.	B.E. (eV)	%Atom	B.E. (eV)	% Atom	B.E. (eV)	% Atom
C1	284.53	38.11	284.6	57.24	284.61	58.82
C2	285.66	29.48	285.75	18.94	285.7	18.91
C3	286.70	10.01	286.7	11.32	286.7	12.27
C4	288.54	8.44	288.72	4.82	288.73	5.81
C5	290.21	13.96	290.59	7.68	290.57	4.20

**Table 6:** Deconvolution of the N1s peak.

	HFM-183		HFM-183+PSS		HFM-183+PSS+Ar	
Component of the N1s Peak	B.E. (eV)	% Atom	B.E. (eV)	% Atom	B.E. (eV)	%Atom
N1	399.21	65.65	399.19	72.37	399.09	68.77
N2	402.24	34.35	401.95	27.63	401.97	31.23

## Supplementary Materials

The following are available online at https://www.mdpi.com/2073-4360/11/10/1689/s1, Figure S1: PVDF configurations. (a) *α* phase and (b) *β* phase, Figure S2: Zoom Raman spectra of (a) HFM-183+PSS and (b) HFM-183+PSS+Ar, Table S1: Peaks in the Raman spectra.
