# Supplementary material for: Morphological, Electrical, and Chemical Characteristics of Poly(sodium 4-styrenesulfonate) Coated PVDF Ultrafiltration Membranes after Plasma Treatment
Source: Polymers (Basel). 2019 Oct 15;11(10):1689. doi: 10.3390/polym11101689 (PMC6836023; doi:10.3390/polym11101689)
Supplement: Supplementary file 1 [file polymers-11-01689-s001.pdf]

polymers

Supplementary Material

Morphological, Electrical and Chemical Characteristics of Poly(sodium 4-styrenesulfonate) Coated PVDF Ultrafiltration Membranes after Plasma Treatment

a)

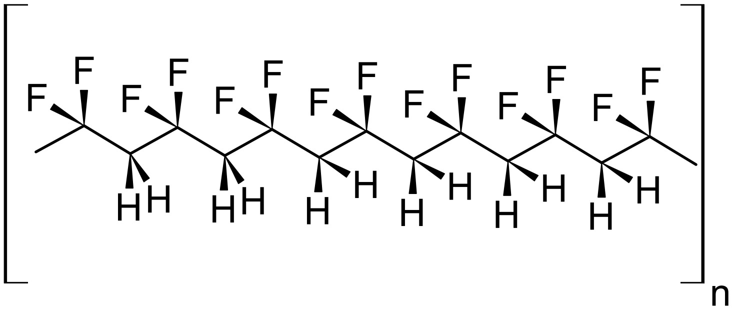

b)

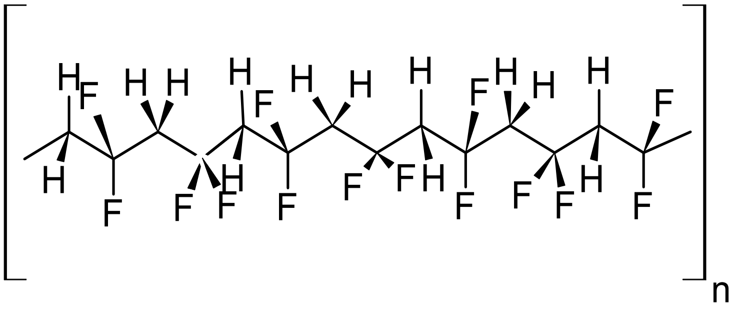

Figure S1. PVDF configurations. **a**) α phase and **b**) β phase.

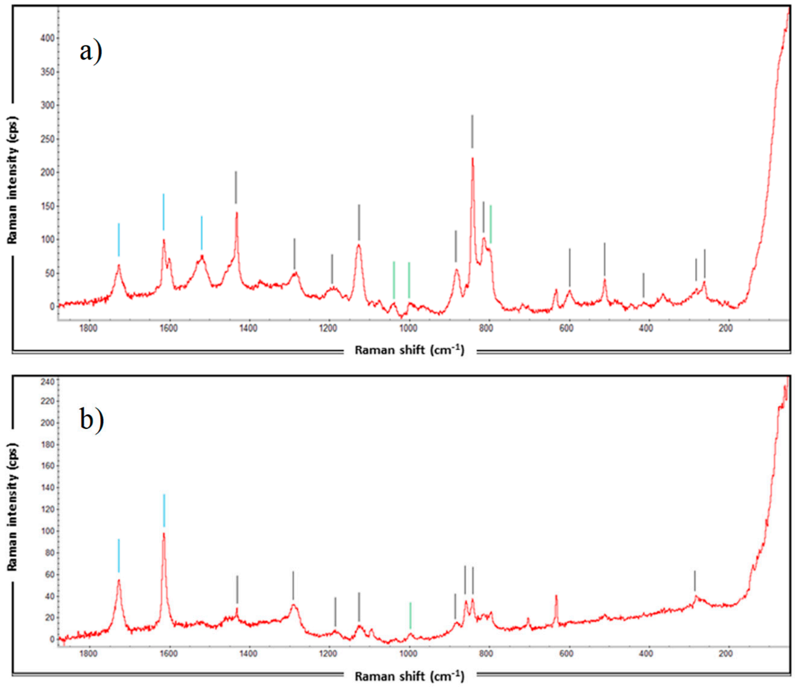

Figure S2. Zoom Raman spectra of **a**) HFM-183+PSS and **b**) HFM-183+PSS+Ar.

Table S1. Peaks in the Raman spectra.

PVDF Film Raman Shift (cm⁻¹)	Peak	HFM-183 Raman Shift (cm⁻¹)	Peak	HFM-183+PSS Raman Shift (cm⁻¹)	Peak	HFM-183+PSS+Ar Raman Shift (cm⁻¹)	Peak
3010	CH asymmetric stretching	3010	CH asymmetric stretching	3010	CH asymmetric stretching	3010	CH asymmetric stretching o
2990	CH symmetric stretching	2990	CH symmetric stretching	3090	C–H aromatic, stretching	3090	C–H aromatic, stretching
1440	CH stretching	1730	C=N	2990	CH symmetric stretching	2990	CH symmetric stretching
1300	CF_2 stretching	1610	NH_3 out of phase bending	2900	C–H aliphatic, stretching	2900	C–H aliphatic, stretching
1200	CF_2 stretching	1520	NH_3 in phase bending	1730	C=N	1730	C=N
1150	C–C stretching	1440	CH stretching	1610	NH_3 out of phase bending	1610	NH_3 out of phase bending
1080	CH bending	1300	CF_2 stretching	1520	NH_3 in phase bending	1520	NH_3 in phase bending
890	CH bending	1200	CF_2 stretching	1450	CH_2 bending	1450	CH_2 bending
850	CH_2 rocking - CF_2 out of phase stretching	1150	C–C	1440	CH stretching	1440	CH stretching
810	CF_2 in phase**	1080	CH bending	1300	CF_2 stretching	1300	CF_2 stretching
610	CF_2 bending – CCC out of phase backbone vitration	1000	NH stretching	1200	CF_2 stretching	1200	CF_2 stretching

510	CF_2 wagging - CF_2 in phase bending	890	CH bending	1150	C–C	1150	C–C
410	CF_2 twisting	850	CH_2 rocking - CF_2 out of phase stretching	1080	CH bending	1080	CH bending
285	CF_2 twisting and wagging	810	CF_2 in phase	1030	$-SO_3^-$ stretching	1030	SO_3 stretching
250	CF_2 twisting and wagging	610	CF_2 bending – CCC out of phase backbone vitration	1005	C–C stretching, p-substituted ring	1000	C–C stretching, p-substituted ring
		510	CF_2 wagging - CF_2 in phase bending	1000	NH stretching	1000	NH stretching
		410	CF_2 twisting	890	CH bending	890	CH bending
		285	CF_2 twisting and wagging	850	CH_2 rocking - CF_2 out of phase stretching	850	CH_2 rocking - CF_2 out of phase stretching
		250	CF_2 twisting and wagging	810	CF_2 in phase	810	CF_2 in phase
				800	C–S stretching	800	C–S stretching
				610	CF_2 bending – CCC out of phase backbone vitration	610	CF_2 bending – CCC out of phase backbone vitration
				600	SO_3 bending	510	CF_2 wagging - CF_2 bending in phase
				510	CF_2 wagging - CF_2 in phase bending	285	CF_2 twisting and wagging
				410	CF_2 twisting		
				285	CF_2 twisting and wagging		
				250	CF_2 twisting and wagging		
